# Environmental safety thresholds for children with asthma symptoms: a prospective study of multitemporal air pollution exposure and longitudinal trajectories

**DOI:** 10.3389/fpubh.2026.1842184

**Published:** 2026-06-05

**Authors:** Ting Chen, Chaoban Wang, Lina Chen

**Affiliations:** 1 Department of Pediatric Pulmonology, West China Second University Hospital, Sichuan University, Chengdu, China; 2 Key Laboratory of Birth Defects and Related Diseases of Women and Children (Sichuan University), Ministry of Education, Chengdu, China; 3 Department of Pediatric Hematology, West China Second University Hospital, Sichuan University, Chengdu, China; 4 NHC Key Laboratory of Chronobiology (Sichuan University), Chengdu, China

**Keywords:** air pollution, childhood asthma, disease trajectories, safety threshold, sulfur dioxide (so2)

## Abstract

**Background:**

Current air quality standards are designed for healthy populations, while their adequacy in protecting children with asthma symptoms remains unclear. We prospectively delineated the effects of air pollution exposures across multiple time windows on the longitudinal trajectories of childhood asthma.

**Methods:**

This 18-month prospective cohort study involved 1,545 high-risk children presenting with asthma symptoms. We utilized latent class growth analysis (LCGA) to identify distinct disease phenotypes, and applied generalized estimating equations (GEE) and LASSO-logistic regression to evaluate the independent associations of individual pollutants across 1-, 3-, and 12-month exposure windows. Finally, Temporal effect decomposition and restricted cubic spline (RCS) models separated long-term cumulative exposures from short-term fluctuations to explore potential safety thresholds.

**Results:**

We identified sustained remission (79.0%) and persistent high-risk (21.0%) trajectories. Multi-temporal modeling revealed that sulfur dioxide (SO2) was uniquely and consistently associated with a higher probability of high-risk trajectory assignment across all windows. Conversely, PM10 and PM2.5 were associated with protective effects, while behavioral heatmaps indicated that high particulate matter (PM) concentrations may have prompted outdoor avoidance and reduced physical activity. High long-term (12-month) SO2 exposure was associated with a baseline risk of persistent symptoms of 25.1% (compared to 10.4% in optimal conditions), whereas short-term (1-month) fluctuations corresponded to an additional 4% absolute risk. The potential safety threshold for long-term SO2 exposure was remarkably low at 8.5 μg/m^3^.

**Conclusion:**

The reference level for long-term SO2 exposure among high-risk children is substantially lower than current conventional standards. Ambient SO2 serves as a critical environmental warning indicator to guide early, individualized preventive interventions in pediatric clinical practice.

## Graphical abstract

**Figure:**
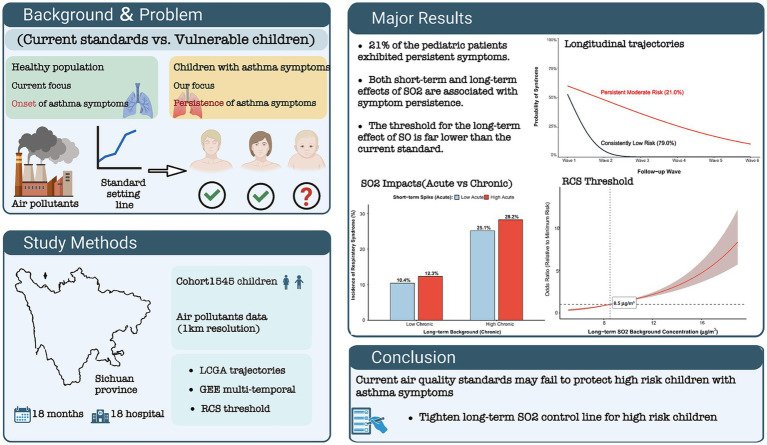

## 1 Introduction

Air pollution is an independent risk factor for the onset and progression of childhood asthma (1–3). To address this public health challenge, the World Health Organization (WHO) and national environmental agencies have established guiding thresholds for core pollutants based on large-scale epidemiological evidence (4). However, these current standards rely primarily on observational data from the general healthy population, mostly adults or healthy children (5–7). This raises a critical clinical question: are these population-based safety limits truly safe for susceptible children who already exhibit asthma symptom?

In pediatric clinical practice, physicians and parents primarily worry whether a susceptible child’s early asthma symptom will become persistent or remit. Although studies link single gaseous pollutants to asthma exacerbations (8), children typically face mixed exposure to multiple pollutants in reality (9, 10). Consequently, amid this multipollutant context, accurately identifying and evaluating the core pollutants with the greatest independent pathogenic weight is essential for effective prevention and control.

Furthermore, current understanding remains limited regarding exposure timing. Previous studies link air pollutant exposure to significantly increased incidence and hospitalization rates for childhood respiratory diseases (11–14). For example, an ecological study in Spain, found that elevated NO2 and O3 concentrations significantly increased the risk of emergency visits and hospitalizations for acute respiratory diseases (especially asthma and bronchiolitis) among children (15). Similarly, a time-series study in China, associated increased NO2 concentrations with a higher risk of childhood asthma hospitalizations (11). However, both these studies and current environmental standards focus predominantly on preventing short-term, acute health endpoints. This approach overlooks the profound impact of long-term, cumulative exposure to low-concentration pollutants on the longitudinal trajectory of the disease’s natural history. It also fails to clearly disentangle the short-term acute triggering from the long-term chronic sensitizing effects of exposure. Disentangling the independent effects of specific pollutants across various exposure durations is a significant challenge. However, such clarity is essential for establishing targeted environmental standards that protect high-risk groups.

To address these gaps regarding thresholds for susceptible populations, multipollutant mixed exposure, and temporal effect decomposition, we prospectively followed 1,545 children with asthma symptoms for 18 months. We aim to track and identify longitudinal phenotypic trajectories of asthma symptom persistence, disentangle the independent pathogenic weights of multiple pollutants across different exposure windows, and establish non-linear safety thresholds for the core pollutants driving disease progression. We hope this study provides a robust, evidence-based foundation for revising targeted public health strategies and home intervention standards.

## 2 Methods

This investigation received financial support from the Major Science and Technology Application Demonstration Project of Chengdu, Sichuan Province, China (Grant No. 2019-YF09-00087-SN) and secured ethical clearance from the Ethics Review Committee of West China Second Hospital, Sichuan University (Approval No. YXKY2020081). The research encompassed a multicenter design involving 18 Grade A tertiary medical institutions distributed across 11 cities within Sichuan Province. Participant recruitment spanned from January 2018 to December 2020, ultimately enrolling 1,545 pediatric subjects following the acquisition of guardian consent.

### 2.1 Study population

Eligibility was restricted to children and adolescents between 0 and 18 years of age presenting to pediatric departments with clinical manifestations of wheezing-associated disorders. Exclusion criteria mandated the removal of participants exhibiting concurrent complications, non-respiratory pathologies, or underlying congenital and genetic anomalies. Prior to enrollment, legal guardians were thoroughly briefed on the study objectives and protocols, providing written informed consent voluntarily.

### 2.2 Inclusion criteria

(1)Pediatric patients aged 0–18 years receiving a physician’s diagnosis of a wheezing-related condition, specifically including clinically verified bronchiolitis, wheezy bronchitis, bronchopneumonia accompanied by wheezing, and bronchial asthma.

(2)Voluntary participation confirmed by guardians who provided documented informed consent.

### 2.3 Exclusion criteria

1. Diagnosis of bronchopulmonary dysplasia or any recognized congenital and genetic syndromes.
2. Presence of structural congenital abnormalities affecting the bronchi or lungs, such as congenital bronchomalacia, stenosis, airway malformations, or pulmonary hypoplasia.
3. Existence of congenital cardiac defects or major vascular anomalies, including congenital heart disease and pulmonary artery sling.
4. Additional comorbidities comprising malnutrition, gastroesophageal reflux disease, obliterative bronchiolitis, or documented immune deficiency states.

To guarantee data integrity, all personnel responsible for data acquisition underwent rigorous standardized training and utilized questionnaires that had been pre-tested for validity and reliability.

### 2.4 Data acquisition and management

#### 2.4.1 Environmental exposures

Ambient air pollution metrics were geospatially linked to the residential coordinates (latitude and longitude) of each enrolled child. These datasets were provided courtesy of Professor Wei Jing (16–20). Monthly mean concentrations were calculated for key pollutants: carbon monoxide (CO), nitrogen dioxide (NO₂), ozone (O₃), sulfur dioxide (SO₂), particulate matter ≤2.5 μm (PM₂.₅), and particulate matter ≤10 μm (PM₁₀). Concentration units were standardized as milligrams per cubic meter (mg/m^3^) for CO and micrograms per cubic meter (μg/m^3^) for remaining species, with a spatial resolution of 1,000 × 1,000 m^2^.

#### 2.4.2 Clinical outcomes

The primary dependent variables focused on the longitudinal progression of clinical symptomatology. These manifestations were meticulously documented during each assessment and primarily covered asthmatic symptoms, specifically including wheezing, coughing, thoracic tightness, and dyspnea.

#### 2.4.3 Covariates

To rigorously control for potential confounding effects in the statistical models, a comprehensive array of parameters was collected as covariates. These included:

1. Demographics and Socioeconomic Status (SES): Age, gender, ethnicity, and parental SES (assessed via maternal and paternal educational attainment).
2. Baseline Clinical History: History of premature birth, and baseline conditions such as allergic rhinitis, eczema, and other allergic conditions.
3. Household Environmental Factors: Distance to the main road, household smoking exposure, recent home renovations, the presence of a fresh air ventilation system, kitchen exhaust hood, and the type of cooking appliance fuel.
4. Lifestyle and Behavioral Metrics: Presence of regular outdoor activities and average outdoor exercise duration.

#### 2.4.4 Recruitment protocol and longitudinal follow-up

Data acquisition was executed by clinically trained physicians and nursing staff during outpatient visits involving participants who satisfied all predefined inclusion criteria. Upon enrollment, a dedicated tracking registry was initiated. Subsequent data collection involved structured follow-ups administered either in-person or via telephone by qualified medical personnel. Guardians were contacted periodically to finalize questionnaire responses, a process sustained until the study’s termination. Specific assessment intervals were scheduled at baseline, followed by evaluations at 3, 6, 9, 12, and 18 months post-enrollment.

### 2.5 Statistical analysis

All statistical analyses were performed using R software (version 4.5.1). A two-sided *p*-value < 0.05 was considered statistically significant. To ensure methodological transparency and mitigate the risk of overfitting, our analytical approach was conceptually structured as a sequential four-step pipeline, with each specific method rigorously chosen to address a distinct, progressive research question:

1. Phenotyping (LCGA): First, to objectively define our target subpopulation, LCGA was utilized to delineate heterogeneous, data-driven longitudinal trajectories of asthma symptoms over the 18-month follow-up.
2. Feature Selection and Association (LASSO and Multivariate Models): Second, to identify the core pollutants independently associated with the high-risk trajectory amid severe environmental multicollinearity, we applied LASSO regression. Crucially, a 10-fold cross-validation was embedded within the LASSO procedure specifically to penalize model complexity and prevent overfitting. The selected features were then validated using multivariate logistic regression.
3. Temporal Decomposition (GEE): Third, having identified SO2 as the prominent environmental factor, we employed a temporal decomposition strategy via GEE models to address when the exposure matters most—statistically disentangling long-term cumulative associations from short-term acute fluctuations.
4. Threshold Determination (RCS): Finally, building directly upon the GEE findings that highlighted the dominance of long-term exposure, we constructed non-linear RCS models to address at what specific concentration the risk accelerates, thereby determining the optimal public health reference threshold.

The detailed parameters and procedures for each of these pipeline steps are outlined below:

(1) Longitudinal Trajectory Identification and Baseline Comparison

Latent Class Growth Analysis (LCGA) was performed using the lcmm package to identify heterogeneous longitudinal trajectories of asthma syndrome across follow-up waves (Waves 1 to 6). We fitted models ranging from 1 to 4 latent classes. The optimal number of classes was determined based on the elbow rule of the Bayesian Information Criterion (BIC) and clinical plausibility (the smallest class proportion > 5%). Based on the optimal model, participants were assigned to specific risk trajectory groups. Baseline characteristics and pollutant exposure levels were summarized as means (standard deviations, SD) for continuous variables and as frequencies (percentages) for categorical variables. Group differences were evaluated using appropriate hypothesis testing (e.g., Student’s t-test or Chi-square test).

(2) Exposure Window Definition and Variable Selection

Three temporal exposure windows for air pollutants were defined: short-term (1-month average before follow-up), medium-term (3-month average), and long-term (12-month average) exposure. To eliminate dimensional effects and ensure comparability of the Odds Ratios (ORs), all pollutant concentrations were Z-score standardized. To address multicollinearity among highly correlated environmental exposures, we applied the Least Absolute Shrinkage and Selection Operator (LASSO) regression with 10-fold cross-validation within each exposure window to select variables significantly associated with the high-risk trajectory. Variables with non-zero coefficients were subsequently incorporated into multivariate logistic regression models, and Variance Inflation Factors (VIF) were calculated.

(3) Temporal Effect Decomposition and Joint Effect Analysis

To disentangle the long-term cumulative associations from the short-term exposure fluctuations of pollutants, we implemented a temporal effect decomposition strategy. The long-term background concentration (12-month average) was used as the long-term baseline, while the short-term fluctuation (1-month average minus the 12-month average) was defined as the short-term acute exposure. Generalized Estimating Equations (GEE) were utilized to evaluate the joint exposure effects, visually represented through a Double Jeopardy Risk Matrix. The predictive performance of various decomposed models was compared using the Area Under the Receiver Operating Characteristic (ROC) Curve (AUC).

(4) Longitudinal Evaluation of Behavioral Avoidance

GEE models with an exchangeable correlation structure were applied to assess the the longitudinal associations between multi-phase air pollution exposures and protective behaviors (e.g., complete outdoor avoidance, reducing outdoor exercise to <3 h), accounting for intra-subject correlation over repeated measurements. Results were visualized using a multiphase behavioral association heatmap.

(5) Non-linear Threshold Exploration.

Restricted Cubic Spline (RCS) models with 3 knots were constructed using the rms package to evaluate the non-linear dose–response relationship between long-term core pollutant exposure and the risk of lower respiratory syndrome, adjusting for short-term fluctuations and other clinical covariates. The concentration corresponding to the minimum predicted risk was set as the reference point (OR = 1). We defined the statistically significant risk-increasing threshold as the concentration at which the lower bound of the 95% Confidence Interval (CI) first exceeded 1.

## 3 Results

### 3.1 Identification of longitudinal trajectories and clinical characteristics

**Figure 1:**
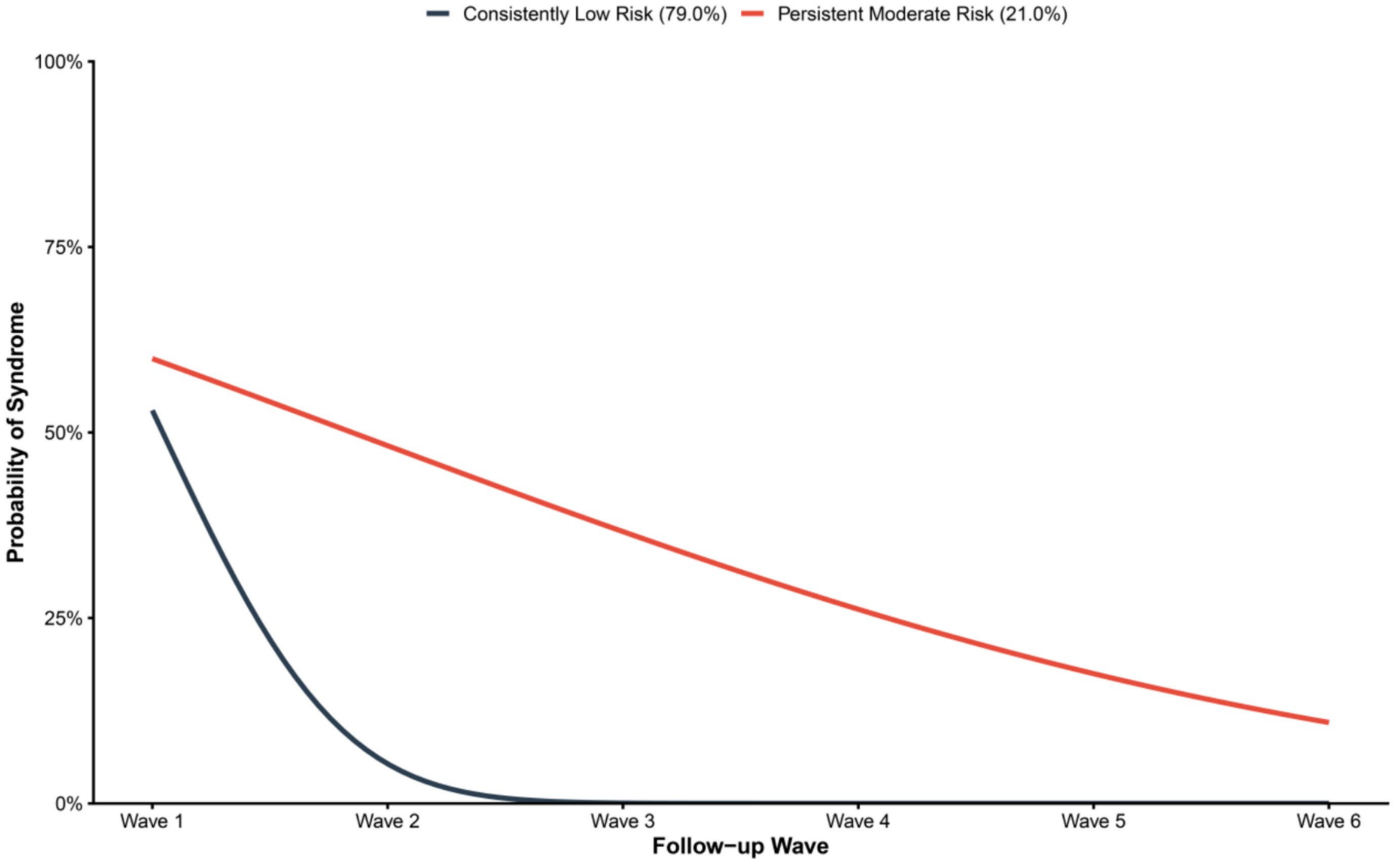
Longitudinal trajectories of lower respiratory syndrome over the 18-month follow-up. Two distinct phenotypic trajectories were identified using latent class growth analysis (LCGA): the consistently low risk group (79.0%, blue line) and the persistent moderate risk group (21.0%, red line). The *x*-axis represents the six sequential follow-up waves, and the *y*-axis indicates the predicted probability of experiencing the syndrome.

To identify heterogeneous patterns of asthma syndrome (≥2 symptoms among cough, wheezing, chest tightness, and dyspnea), we applied LCGA across six follow-up waves. Although the BIC decreased for models with up to four classes (Supplementary Figure S1), three- and four-class models yielded unreliably small subgroups (<1%). Therefore, we selected the robust two-class model. It delineated a Consistently Low Risk trajectory (79.0%, *n* = 1,221), maintaining a stable, low symptom probability, and a Persistent Moderate Risk trajectory (21.0%, *n* = 324), exhibiting an elevated, fluctuating risk throughout the 18-month follow-up (Figure 1).

Table 1 summarizes the demographic, clinical, and environmental exposure characteristics of the study population at baseline, stratified by the two trajectory phenotypes. Compared to the low-risk group, children in the Persistent Moderate Risk group were younger (3.17 ± 2.54 vs. 3.91 ± 2.88 years, *p* < 0.001), predominantly male (73% vs. 63%, *p* = 0.001), and had lower parental education (*p* = 0.005 for fathers; *p* = 0.008 for mothers). Notably, the two groups showed no significant differences in traditional intrinsic risk factors, including a history of premature birth, household smoking exposure, and baseline allergic conditions (e.g., allergic rhinitis, eczema, and other allergies).

**Table 1:** Baseline characteristics and environmental exposures of the study population according to longitudinal trajectories.

Characteristics	Persistent moderate risk (21.0%)	Consistently low risk (79.0%)	*p*-value^a^
	*N* = 324	*N* = 1,221	
Gender, *n* (%)			0.001
Male	235 (73)	766 (63)	
Female	89 (27)	455 (37)	
Age, mean (SD), years	3.17 (2.54)	3.91 (2.88)	<0.001
Father’s education, *n* (%)			0.005
High school or below	200 (62)	648 (53)	
Above high school	124 (38)	573 (47)	
Mother’s education, *n* (%)			0.008
High school or below	211 (65)	695 (57)	
Above high school	113 (35)	526 (43)	
Ethnicity, *n* (%)			0.589
Non-Han	5 (1.5)	15 (1.2)	
Han	319 (98)	1,206 (99)	
History of premature birth, *n* (%)			0.268
No	303 (94)	1,119 (92)	
Yes	21 (6.5)	102 (8.4)	
Outdoor activities, *n* (%)			0.078
No	238 (73)	835 (68)	
Yes	86 (27)	386 (32)	
Household smoking exposure, *n* (%)			0.484
No	167 (52)	656 (54)	
Yes	157 (48)	565 (46)	
Outdoor exercise duration, *n* (%)			0.583
≥ 3 h	99 (31)	354 (29)	
< 3 h	225 (69)	867 (71)	
Recent home renovation, *n* (%)			0.208
No	303 (94)	1,163 (95)	
Yes	21 (6.5)	58 (4.8)	
Distance to main road, *n* (%)			0.015
≤ 100 m	107 (33)	494 (40)	
> 100 m	217 (67)	727 (60)	
Fresh air ventilation system, *n* (%)			0.434
No	278 (86)	1,026 (84)	
Yes	46 (14)	195 (16)	
Kitchen exhaust hood, *n* (%)			0.289
No	57 (18)	247 (20)	
Yes	267 (82)	974 (80)	
Cooking appliance fuel, *n* (%)			0.185
Electric stove	21 (6.5)	57 (4.7)	
Non-electric stove	303 (94)	1,164 (95)	
Allergic rhinitis, *n* (%)			0.586
No	228 (70)	840 (69)	
Yes	96 (30)	381 (31)	
Eczema, *n* (%)			0.741
No	171 (53)	657 (54)	
Yes	153 (47)	564 (46)	
Other allergic conditions, *n* (%)			0.164
No	273 (84)	1,065 (87)	
Yes	51 (16)	156 (13)	
Air pollution^b^			
SO2_1m, mean (SD), μg/m^3^	8.77 (1.60)	8.40 (1.55)	<0.001
NO2_1m, mean (SD), μg/m^3^	27 (7)	28 (8)	0.022
PM2.5_1m, mean (SD), μg/m^3^	34 (7)	34 (6)	0.606
PM10_1m, mean (SD), μg/m^3^	54 (9)	54 (9)	0.583
CO_1m, mean (SD), μg/m^3^	0.76 (0.06)	0.76 (0.07)	0.358
O3_1m, mean (SD), μg/m^3^	97 (8)	99 (12)	<0.001
SO2_3m, mean (SD), μg/m^3^	8.92 (1.63)	8.53 (1.55)	<0.001
NO2_3m, mean (SD), μg/m^3^	28 (7)	29 (8)	0.014
PM2.5_3m, mean (SD), μg/m^3^	36 (7)	37 (6)	0.023
PM10_3m, mean (SD), μg/m^3^	56 (9)	57 (9)	0.016
CO_3m, mean (SD), μg/m^3^	0.78 (0.06)	0.78 (0.06)	0.879
O3_3m, mean (SD), μg/m^3^	94 (8)	96 (9)	<0.001
SO2_12m, mean (SD), μg/m^3^	9.37 (1.93)	8.84 (1.74)	<0.001
NO2_12m, mean (SD), μg/m^3^	29 (7)	30 (8)	0.014
PM2.5_12m, mean (SD), μg/m^3^	37.2 (6.4)	38.4 (5.4)	<0.001
PM10_12m, mean (SD), μg/m^3^	58 (8)	60 (8)	<0.001
CO_12m, mean (SD), μg/m^3^	0.80 (0.06)	0.80 (0.06)	0.569
O3_12m, mean (SD), μg/m^3^	89.7 (5.4)	89.8 (4.8)	0.13

CO, carbon monoxide; NO2, nitrogen dioxide; O3, ozone; PM2.5, fine particulate matter; PM10, inhalable particulate matter; SO2, sulfur dioxide.

a Pearson’s Chi-squared test; Wilcoxon rank sum test; Fisher’s exact test.

b The suffixes 1 m, 3 m, and 12 m denote the 1-month, 3-month, and 12-month temporal exposure windows prior to baseline, respectively.

Crucially, the two phenotypic groups exhibited stark contrasts in their ambient air pollution exposure levels. Children in the Persistent Moderate Risk trajectory were exposed to significantly higher levels of SO2 across all temporal windows (short-term 1-month, medium-term 3-month, and long-term 12-month). In contrast, the concentrations of NO2, O3, PM2.5, and PM10 demonstrated a statistically significant inverse association with risk. CO exposure showed no significant differences between the two groups.

### 3.2 Independent associations of multi-phase pollutant exposures

To systematically identify risk factors for the Persistent Moderate Risk trajectory, we initially conducted a univariate logistic regression screening (Supplementary Table S1). Given the severe multicollinearity among co-existing ambient air pollutants, we applied LASSO regression with 10-fold cross-validation across the 1-, 3-, and 12-month exposure windows for feature reduction and variable selection (Supplementary Figure S2). Variables with non-zero coefficients in the optimized LASSO models were subsequently incorporated into the final multivariate logistic regression analysis (Table 2). Variance Inflation Factor (VIF) diagnostics confirmed the absence of severe multicollinearity across all three temporal models (Supplementary Figure S3).

**Table 2:** Multivariate logistic regression models identifying independent risk factors for the persistent moderate risk trajectory across different exposure windows.

Variables	1-month exposure model	*p*-value	3-month exposure model	*p*-value	12-month exposure model	*p*-value
Demographics						
Age (per 1-year increase)	0.89 (0.84–0.94)	<0.001	0.88 (0.83–0.93)	<0.001	0.89 (0.84–0.94)	<0.001
Sex (Female vs. Male)	0.66 (0.50–0.87)	0.004	0.66 (0.50–0.87)	0.004	0.66 (0.50–0.87)	0.003
Ethnicity (Han vs. Non-Han)	—	—	—	—	1.70 (0.58–5.71)	0.356
Father’s education (> vs. ≤ High school)	0.92 (0.62–1.36)	0.687	0.90 (0.61–1.32)	0.588	0.88 (0.59–1.29)	0.504
Mother’s education (> vs. ≤ High school)	0.84 (0.57–1.24)	0.377	0.85 (0.57–1.26)	0.409	0.84 (0.57–1.24)	0.378
Clinical characteristics						
History of premature birth (Yes vs. No)	0.66 (0.39–1.07)	0.104	0.65 (0.39–1.06)	0.098	0.68 (0.40–1.09)	0.126
Allergic rhinitis (Yes vs. No)	—	—	1.14 (0.84–1.54)	0.393	—	—
Other allergic conditions (Yes vs. No)	1.36 (0.95–1.93)	0.092	1.37 (0.94–1.96)	0.094	1.33 (0.93–1.90)	0.115
Household environment						
Outdoor activities (Yes vs. No)	0.82 (0.62–1.09)	0.182	0.82 (0.62–1.09)	0.181	0.84 (0.63–1.11)	0.22
Distance to main road (> 100 vs. ≤ 100 m)	1.47 (1.13–1.93)	0.005	1.48 (1.14–1.94)	0.004	1.45 (1.11–1.90)	0.006
Recent home renovation (Yes vs. No)	1.35 (0.77–2.28)	0.281	1.37 (0.78–2.32)	0.257	1.32 (0.76–2.23)	0.314
Kitchen exhaust hood (Yes vs. No)	1.51 (1.08–2.15)	0.019	1.48 (1.06–2.09)	0.025	1.51 (1.08–2.14)	0.019
Air pollutant exposures (per 1 SD)						
SO2	1.21 (1.07–1.37)	0.003	1.18 (1.04–1.35)	0.012	1.23 (1.07–1.42)	0.004
CO	—	—	—	—	—	—
NO2	0.88 (0.76–1.02)	0.083	—	—	—	—
PM2.5	—	—	0.83 (0.72–0.95)	0.008	—	—
PM10	—	—	—	—	0.84 (0.73–0.98)	0.029
O3	0.78 (0.68–0.89)	<0.001	0.75 (0.65–0.86)	<0.001	0.94 (0.82–1.07)	0.321

CI, confidence interval; NO2, nitrogen dioxide; O3, ozone; OR, odds ratio; PM2.5, fine particulate matter; PM10, inhalable particulate matter; SD, standard deviation; SO2, sulfur dioxide.

**Figure 2:**
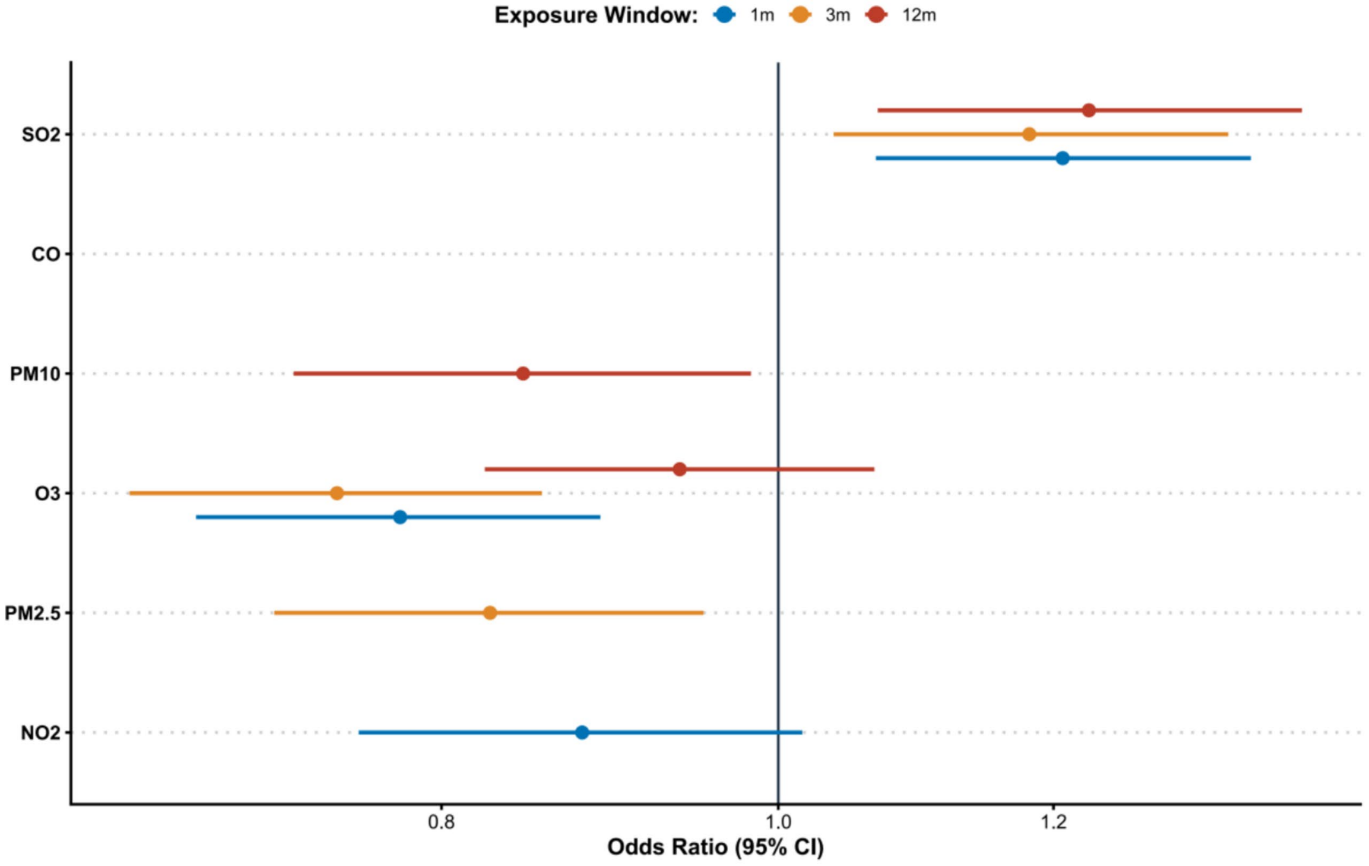
Associations between multi-phase air pollutant exposures and the risk of the persistent moderate wheezing trajectory. The forest plot displays the adjusted odds ratios (ORs) and 95% confidence intervals (CIs) associated with a 1-standard deviation (SD) increase in the concentration of each pollutant. Exposures were evaluated across three temporal windows: short-term (1-month, blue), medium-term (3-month, yellow), and long-term (12-month, red).

After adjusting for confounders, SO2 emerged as the only air pollutant that consistently and significantly associated with an increased risk of the persistent wheezing trajectory across all three exposure windows (Figure 2). Specifically, in the long-term (12-month) window, each 1-standard-deviation (SD) increase in SO2 concentration significantly elevated the odds of entering the high-risk group by 22.9% (OR = 1.23, 95% CI: 1.07–1.42, *p* < 0.01). Similarly robust risk-increasing effects were observed for medium-term 3-month (OR = 1.181, 95% CI: 1.037–1.347) and short-term 1-month (OR = 1.207, 95% CI: 1.067–1.368) exposures. These results underscore the dominant and independent contribution of SO2 during both the short-term exposure and long-term cumulative phases of childhood asthma syndrome.

In stark contrast to the robust pathogenic effects of SO2, multivariate models across multiple exposure windows revealed a counterintuitive, statistically significant negative association between exposure to particulate matter (PM2.5 and PM10) and NO2 and the risk of asthma exacerbation. Given the well-established biological mechanisms by which particulates induce airway inflammation, we hypothesized that this apparent protective effect is not a true physiological benefit, but may rather stems from a classic epidemiological behavioral avoidance effect. Using longitudinal Generalized Estimating Equation (GEE) models, we evaluated the impact of multi-phase pollutant concentrations on the protective behaviors of the patients’ families (Supplementary Figure S4). The multi-phase behavioral association heatmap confirmed that high concentrations of PM2.5 and PM10 in the short- and medium-term (1- and 3-month windows) significantly triggered outdoor avoidance behaviors. However, we must emphasize that this behavioral explanation remains speculative, primarily because our study did not capture data on indoor air quality. Increased time spent indoors may expose susceptible individuals to different, unmeasured indoor pollutants or allergens, acting as a significant confounder in this observed association.

Notably, high SO2 exposure did not trigger similar protective behaviors. Compared to visibly apparent particulate smog, the insidious and invisible nature of gaseous SO2 likely led to a lack of parental vigilance, resulting in a failure to adopt timely avoidance measures.

### 3.3 Temporal effect decomposition and non-linear threshold for long-term exposure

To accurately disentangle the short-term exposure fluctuations and long-term cumulative associations of SO2, we first evaluated the predictive performance of different temporal decomposition combinations using ROC curve analysis (Supplementary Figure S5). The combination of the 1-month acute fluctuation versus the 12-month chronic background yielded the highest Area Under the Curve (AUC = 0.654), outperforming other temporal models. Consequently, this optimal combination was selected for the final decomposition analysis.

The Generalized Estimating Equation (GEE) decomposition model revealed that both long-term background exposure and short-term acute fluctuations independently increased the risk of the persistent moderate risk trajectory. However, long-term cumulative exposure demonstrated a substantially stronger association (OR = 1.30, 95% CI: 1.23–1.36, *p* < 0.001 per 1 SD increase) compared to the short-term fluctuation (OR = 1.04, 95% CI: 1.00–1.08, *p* = 0.041).

**Figure 3:**
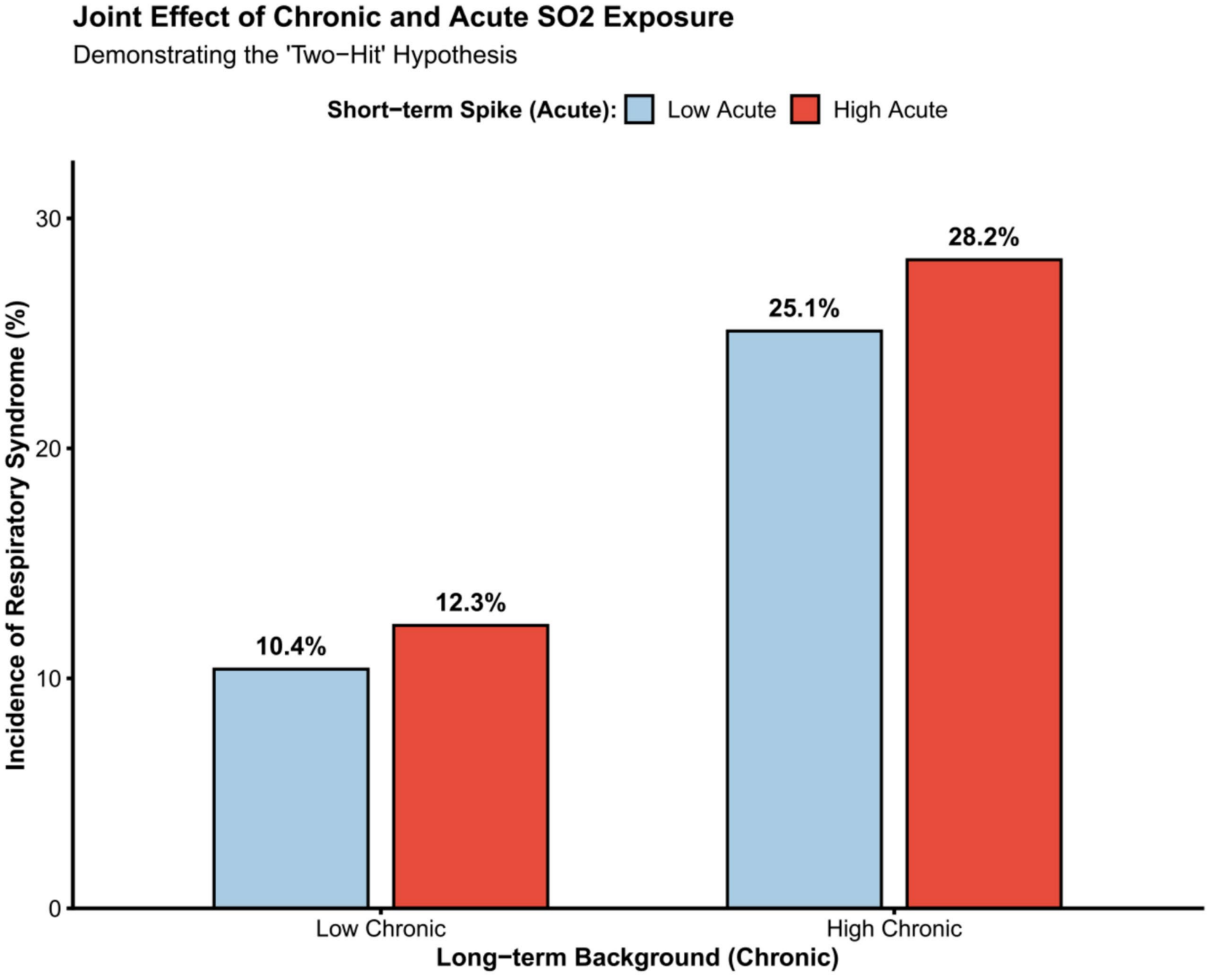
Joint effect of chronic and acute SO_2_ exposures on the incidence of the persistent wheezing trajectory. The bar chart displays the absolute incidence rates (%) of the high-risk syndrome across four distinct exposure combinations. Chronic refers to the long-term (12-month) background SO_2_ level, while acute represents the short-term (1-month) SO_2_ fluctuation. Shifting to a high chronic background dramatically elevates the baseline incidence from 10.4 to 25.1%, even in the absence of acute spikes (isolated sensitization). The superimposition of a high acute spike on a high chronic background results in the highest overall incidence (28.2%).

This dominance of chronic sensitization is further illustrated by the joint effect analysis (Figure 3, Table 3). Compared to the optimal baseline scenario, which had an incidence rate of 10.4%, isolated short-term acute spikes on a low background only marginally increased the incidence to 12.3%. In stark contrast, shifting to a high long-term chronic background dramatically elevated the baseline incidence to 25.1%, even in the absence of acute spikes. These findings indicated that long-term accumulation is a primary factor associated with the disease trajectory, while short-term fluctuations may act as secondary exacerbating factors.

**Table 3:** Independent and joint effects of decomposed short-term acute and long-term chronic SO2 exposures on the risk of persistent wheezing trajectory.

Panel A: Independent effects (GEE decomposition model)*		Adjusted OR (95% CI)	*p*-value
Long-term background exposure (Chronic, 12-month avg.)	—	1.30 (1.23–1.36)	<0.001
Short-term acute fluctuation (Acute, 1-month vs. 12-month)	—	1.04 (1.00–1.08)	0.041
Panel B: Joint effects†	*N*	Incidence (%)	*p*-value
Low chronic and low acute (optimal baseline)	2,203	10.40%	—
Low chronic and high acute (isolated trigger)	3,140	12.30%	—
High chronic and low acute (isolated sensitization)	1,695	25.10%	—
High chronic and high acute (double jeopardy)	1,197	28.20%	—

CI, confidence interval; GEE, generalized estimating equation; OR, odds ratio; SD, standard deviation; SO2, sulfur dioxide.

*Panel A presents the independent pathogenic weights derived from the Generalized Estimating Equation (GEE) decomposition model. The Odds Ratios (ORs) and 95% Confidence Intervals (CIs) represent the risk associated with a 1-standard-deviation (SD) increase in the respective SO2 exposure component.

^†^Panel B displays the actual incidence rates of the “Persistent Moderate Risk” trajectory under different exposure combinations. High and Low levels are defined based on whether the Z-score of the respective decomposed SO2 component is greater than or less than zero.

**Figure 4:**
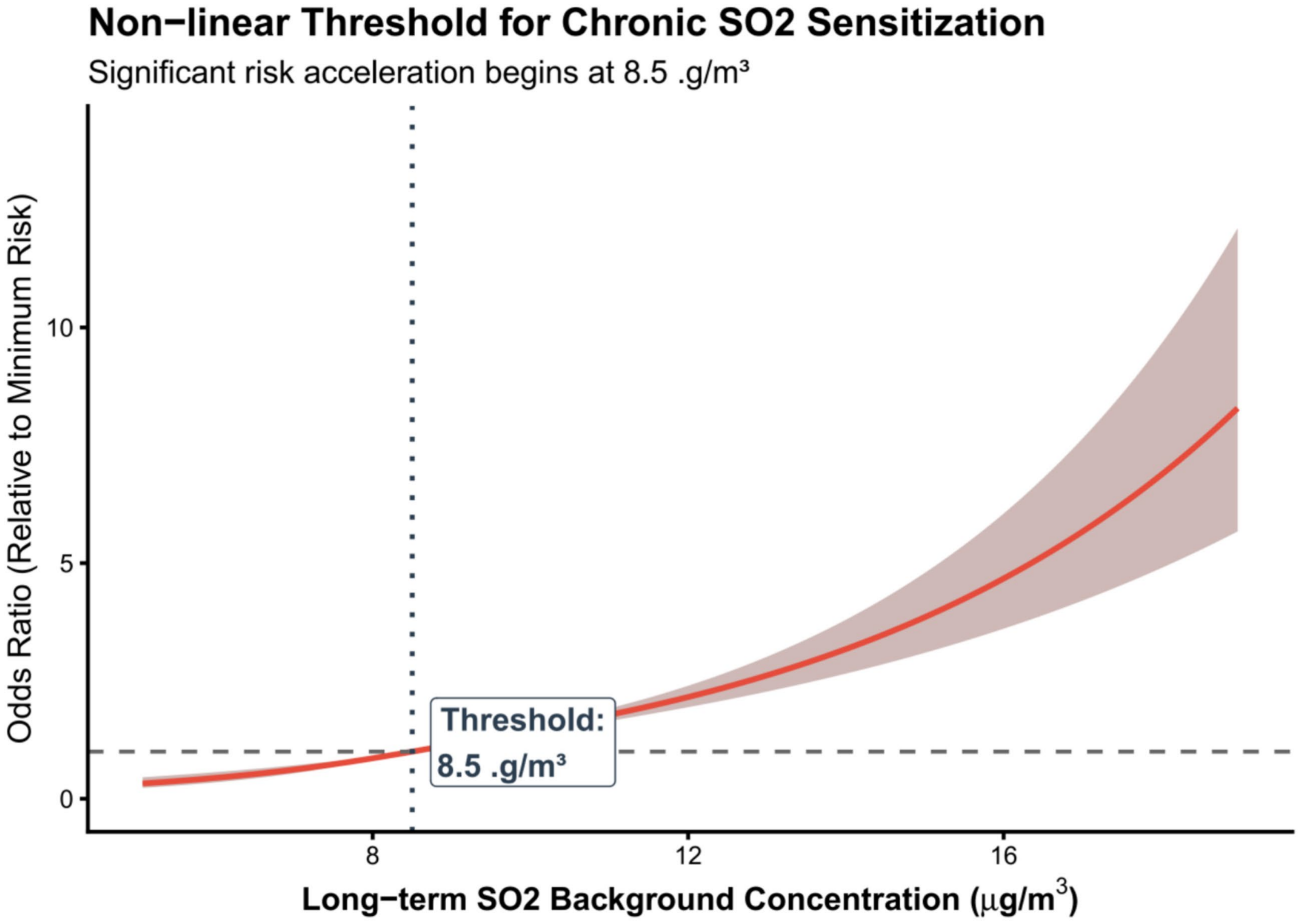
Non-linear dose–response relationship between long-term chronic SO_2_ exposure and the risk of the persistent wheezing trajectory. The solid line represents the estimated odds ratios (ORs) derived from the restricted cubic spline (RCS) model, with the shaded area indicating the 95% confidence intervals (CIs). The model was adjusted for short-term acute SO_2_ fluctuations, age, and other clinical covariates. The reference point (OR = 1.0) was set at the concentration associated with the minimum predicted risk. A highly significant non-linear risk-increasing trend was observed (Overall *p* < 0.001). The lower bound of the 95% CI first significantly exceeds 1.0 at a long-term SO_2_ concentration of 8.5 μμg/m^3^ (indicated by the vertical dashed line). This inflection point defines a critically low safety threshold for chronic sensitization, beyond which the disease risk surges exponentially.

Given the dominant role of long-term SO2 exposure, we employed an RCS model to characterize its non-linear dose–response relationship with the high-risk trajectory and to identify a potential safety threshold (Figure 4). After adjusting for short-term acute fluctuations and clinical covariates, the RCS curve exhibited a highly significant non-linear risk-increasing trend (Total *p* < 0.001). Setting the concentration associated with the minimum predicted risk as the reference (OR = 1.0), we identified a critically low chronic sensitization threshold. The lower bound of the 95% CI first significantly exceeded 1.0 at a long-term SO2 concentration of exactly 8.5 μg/m^3^. Beyond this inflection point, the risk of developing a persistent wheezing trajectory surged exponentially.

## 4 Discussion

Through an 18-month multicenter prospective follow-up, this study investigated the complex associations between multi-temporal air pollution exposure and the longitudinal evolution of asthma symptoms in high-risk children. The primary findings are as follows: First, SO2 exposure is a critical environmental risk factor associated with a higher likelihood of high-risk children into a persistent symptom trajectory. Second, temporal exposure decomposition suggests that long-term cumulative exposure may play a pivotal role in persistent symptoms, whereas short-term fluctuations act as secondary exacerbating factors. Concurrently, data analysis indicates that specific avoidance behaviors may interfere with evaluating the true associations of exposure with certain visible pollutants. Furthermore, we identified a notably low reference SO2 exposure concentration (8.5 μg/m^3^) for asthma-susceptible children.

Current global ambient air quality standards (such as the WHO guidelines and various national annual target values, SO2 exposure concentration typically set between 20 and 60 μg/m^3^) rely heavily on epidemiological evidence derived from healthy populations (21). However, these standards may not adequately protect susceptible children. The research data demonstrate a non-linear dose–response relationship between long-term SO2 exposure and the risk of a persistent wheezing trajectory. The inflection points of 8.5 μg/m^3^ observed in the model suggests that once exposure concentrations exceed this threshold, the risk of persistent symptoms exacerbation exhibits a significant upward trend. This finding aligns with the supra-linear risk at low concentrations observed in several recent large-scale cohorts (22–25). It provides a potential explanation for why some patients continue to experience prolonged symptoms in environments meeting acceptable air quality standards.

Current epidemiological evidence concerning air pollution and pediatric respiratory health diverges methodologically: most time-series or case-crossover studies focus on the association between short-term pollution peaks and increased asthma emergency visits or hospitalization rates (14, 26–28); conversely, long-term birth cohort studies primarily explore the impact of cumulative early-life exposure on initial incidence rates (29–32). In contrast, few studies have quantitatively separated and compared the effects of these two time-scales within the same clinical cohort of individuals with asthma symptoms.

Our data analysis indicates that the positive association between long-term, low-dose cumulative SO2 exposure and a persistent high-risk trajectory is statistically greater than that of 1-month short-term acute fluctuations. As a highly water-soluble gaseous pollutant that readily penetrates the lower respiratory tract, SO2 can still induce significant systemic toxicity even at low concentrations far below current WHO guidelines (33, 34). In the context of the Developmental Origins of Health and Disease hypothesis (35), higher background concentrations of SO2 may induce oxidative stress, thereby affecting the epigenetic modifications of specific genes, such as those related to inflammation and airway hyperresponsiveness. This process could subsequently drive the immune system toward a biased state (36–38). In this cohort, long-term chronic SO2 exposure demonstrated a pronounced association with persistent risk. While our observational design precludes causal conclusions, based on external experimental evidence, we speculate that it may have altered the children’s biological susceptibility to some extent (39). This potential chronic sensitization mechanism may help explain why some patients maintain a persistent symptom trajectory even in the absence of short-term pollution peak triggers (40).

In the multi-pollutant models, fine particulate matter (PM2.5 and PM10) exhibited an unexpected negative association with the risk of asthma exacerbation. Combined with the results of the longitudinal generalized estimating equation (GEE) analysis, we postulate that this phenomenon may partially stem from a behavioral avoidance effect (41). High concentrations of visible particulate pollution more readily trigger parental vigilance, prompting avoidance measures such as reducing children’s outdoor activities. However, it is crucial to emphasize that this behavioral mechanism remains purely a speculative hypothesis rather than a definitive conclusion.

To avoid overinterpretation, several alternative explanations must be explicitly considered. First, there is a substantial risk of exposure misclassification; our reliance on fixed-site residential monitoring objectively overestimates the patients’ actual inhaled doses when they remain indoors during heavy smog (42). Second, this result may be heavily influenced by residual confounding, as increased time spent indoors exposes susceptible children to unmeasured indoor pollutants or allergens that could severely distort the observed associations. Finally, issues with model specification cannot be ruled out; despite acceptable multicollinearity diagnostics, complex interactions among co-existing pollutants could still generate statistical artifacts. Although this behavioral hypothesis requires future validation using individual wearable devices, it indirectly highlights the potential insidious risk of gaseous SO2: because it is colorless, transparent, and unlikely to trigger visual alerts, the public often struggles to adopt timely, targeted outdoor avoidance measures.

Currently, annual SO2 concentration standards in some countries typically range between 20 and 60 μg/m^3^ (43–45). The non-linear threshold model analysis in this study suggests that when evaluating risk for susceptible children with airway hyperresponsiveness using conventional standards based on the general population, the potential limitations of these standards must be considered.

Nevertheless, given the exploratory nature of our findings and and this specific geographical context, future external validation in diverse, large-scale cohorts across broader climatic and environmental heterogeneities is essential before these estimates can be confidently translated into definitive personalized environmental management strategies or formal public health policies.

## 5 Limitations

This study has certain limitations. First, as an observational epidemiological cohort, this study cannot establish causality. Despite the application of rigorous restricted cubic spline (RCS) models and temporal decomposition analysis, we cannot directly confirm the micro-causal mechanisms underlying the 8.5 μg/m^3^ concentration inflection point, and any proposed biological pathways discussed herein remain purely speculative. Future research must integrate toxicology or multi-omics technologies to further investigate the potential pathways through which long-term, low-dose SO2 exposure affects the airway immune microenvironment.

Second, the validity of our air pollution exposure measurement warrants careful consideration. Our assessment relies on residential geospatial data at a 1 km resolution. While this provides robust ambient estimates, it cannot perfectly represent true individual-level inhalation doses, as it inherently does not account for children’s specific mobility patterns, the significant proportion of time spent indoors, or complex micro-environmental variations. This gap introduces an inevitable risk of exposure misclassification bias. However, in spatial epidemiology, such misclassification is generally non-differential and typically biases the observed associations toward the null. This suggests that the true long-term associations between SO2 and persistent asthma risk could potentially be even more pronounced than our models indicate. Furthermore, due to this macro-environmental measurement limitation, the observed 8.5 μg/m^3^ inflection point should not be interpreted as a precise individual biological threshold, but rather as an ambient public health reference level. The lack of individual-level monitoring via GPS trajectories or wearable sensors also means our earlier discussion regarding particulate-induced avoidance behaviors is presented strictly as a hypothesis.

Third, our study may not have fully accounted for uncontrolled confounders, such as indoor air quality, maternal smoking, in-utero tobacco exposure, and the baseline phenotypic heterogeneity since children with established asthma were not excluded. Finally, this study cohort is predominantly concentrated in southwestern China. The specific geographical and climatic characteristics, as well as the emission structure of this region, may affect the generalizability of the findings. Future external validation of this reference concentration is required in cohorts with broader geographical and climatic heterogeneity.

## 6 Conclusion

In conclusion, long-term cumulative SO2 exposure is significantly and positively associated with the persistence of asthma symptoms in susceptible children. The exposure inflection point (8.5 μg/m^3^) evaluated by the non-linear model falls below current conventional public health environmental standards. However, this result should be regarded as an exploratory estimate that is currently limited to our specific regional context. This study suggests that for high-risk pediatric populations with early asthma symptoms, ambient gaseous SO2 is an epidemiological risk factor warranting close attention. Incorporating such environmental indicators into pediatric clinical practice and public health management may facilitate the development of more targeted early interventions and personalized environmental management strategies for patients. Ultimately, to ensure robust external validity, future studies in regions with diverse geographic and climatic characteristics are required before these local findings can guide widespread environmental policy revisions.

## Data Availability

The raw data supporting the conclusions of this article will be made available by the authors, without undue reservation.
