# Supplementary material for: Environmental safety thresholds for children with asthma symptoms: a prospective study of multitemporal air pollution exposure and longitudinal trajectories
Source: Front Public Health. 2026 Jun 5;14:1842184. doi: 10.3389/fpubh.2026.1842184 (PMC13279215; doi:10.3389/fpubh.2026.1842184)
Supplement: Supplementary file 1 [file Supplementary_file_1.docx]

| **Table S1. Univariate Logistic Regression Analysis of Factors Associated with the Persistent Moderate Risk trajectory.** | | |
| --- | --- | --- |
| **Variables** | **OR (95% CI)** | ***P* value** |
| Demographics |  |  |
| Age (per 1-year increase) | 0.90 (0.86–0.95) | <0.001 |
| Sex (Female vs. Male) | 0.64 (0.48–0.83) | 0.001 |
| Ethnicity (Han vs. Non-Han) | 0.79 (0.30–2.46) | 0.657 |
| Father's education (> vs. ≤ High school) | 0.70 (0.55–0.90) | 0.005 |
| Mother's education (> vs. ≤ High school) | 0.71 (0.55–0.91) | 0.008 |
| Clinical & Household Characteristics |  |  |
| History of premature birth (Yes vs. No) | 0.76 (0.46–1.21) | 0.27 |
| Allergic rhinitis (Yes vs. No) | 0.93 (0.71–1.21) | 0.586 |
| Eczema (Yes vs. No) | 1.04 (0.81–1.33) | 0.741 |
| Other allergic conditions (Yes vs. No) | 1.27 (0.90–1.79) | 0.165 |
| Distance to main road (> 100 vs. ≤ 100 m) | 1.38 (1.07–1.79) | 0.015 |
| Household smoking exposure (Yes vs. No) | 1.09 (0.85–1.40) | 0.484 |
| Recent home renovation (Yes vs. No) | 1.39 (0.81–2.29) | 0.21 |
| Fresh air ventilation system (Yes vs. No) | 0.87 (0.61–1.22) | 0.435 |
| Kitchen exhaust hood (Yes vs. No) | 1.19 (0.87–1.65) | 0.289 |
| Cooking appliance (Non-electric vs. Electric) | 0.71 (0.43–1.21) | 0.187 |
| Outdoor activities (Yes vs. No) | 0.78 (0.59–1.02) | 0.079 |
| Outdoor exercise duration (< 3 vs. ≥ 3 hours) | 0.93 (0.71–1.22) | 0.583 |
| Air Pollutant Exposures (per 1 SD increase) |  |  |
| SO2 |  |  |
| 1-month window | 1.24 (1.10–1.39) | <0.001 |
| 3-month window | 1.25 (1.12–1.41) | <0.001 |
| 12-month window | 1.31 (1.17–1.47) | <0.001 |
| NO2 |  |  |
| 1-month window | 0.84 (0.74–0.95) | 0.008 |
| 3-month window | 0.84 (0.73–0.95) | 0.006 |
| 12-month window | 0.84 (0.73–0.95) | 0.006 |
| PM2.5 |  |  |
| 1-month window | 0.96 (0.85–1.09) | 0.545 |
| 3-month window | 0.86 (0.76–0.97) | 0.015 |
| 12-month window | 0.82 (0.73–0.92) | 0.001 |
| PM10 |  |  |
| 1-month window | 0.94 (0.83–1.06) | 0.32 |
| 3-month window | 0.84 (0.74–0.94) | 0.004 |
| 12-month window | 0.79 (0.70–0.89) | <0.001 |
| O3 |  |  |
| 1-month window | 0.77 (0.68–0.88) | <0.001 |
| 3-month window | 0.80 (0.70–0.91) | <0.001 |
| 12-month window | 0.97 (0.86–1.10) | 0.641 |
| CO |  |  |
| 1-month window | 1.03 (0.91–1.16) | 0.673 |
| 3-month window | 0.97 (0.85–1.09) | 0.595 |
| 12-month window | 0.95 (0.84–1.08) | 0.434 |
| Abbreviations: CI, confidence interval; CO, carbon monoxide; NO2, nitrogen dioxide; O3, ozone; OR, odds ratio; PM2.5, fine particulate matter; PM10, inhalable particulate matter; SD, standard deviation; SO2, sulfur dioxide. | | |


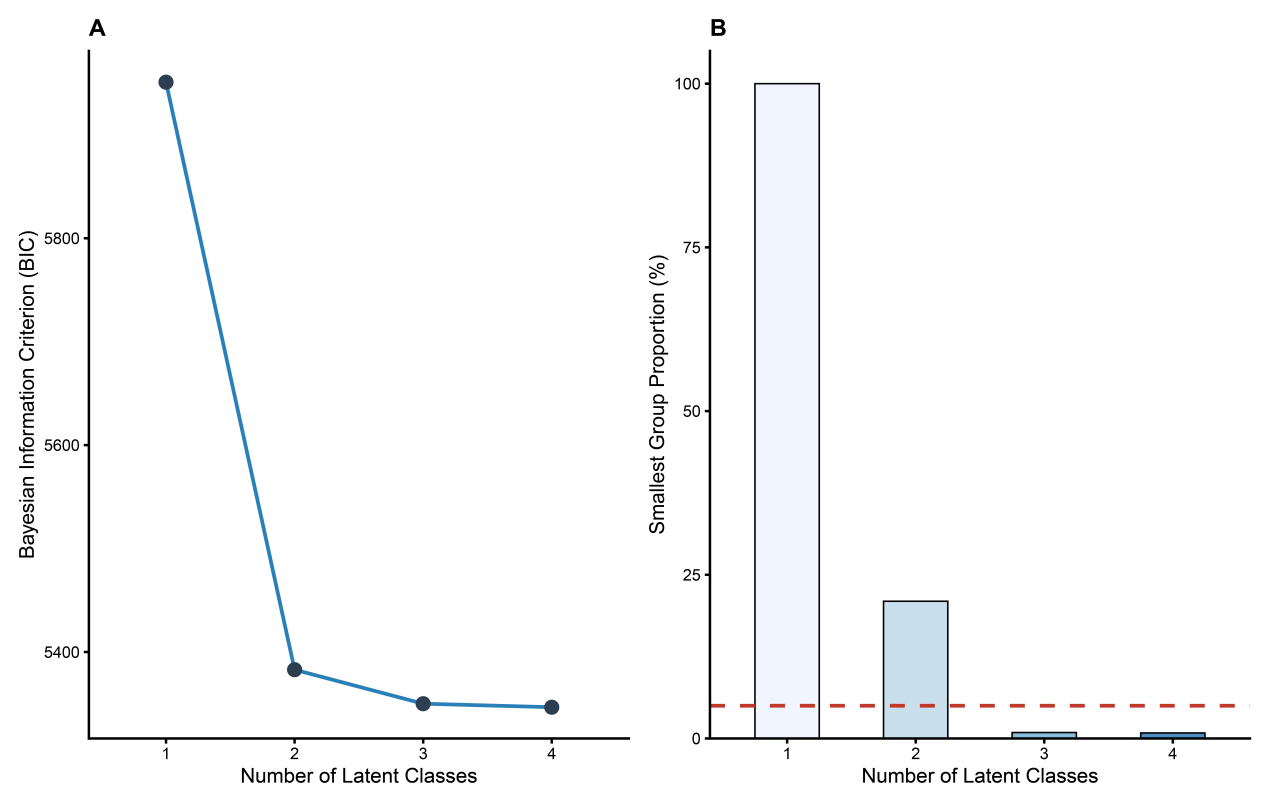


**Figure S1. Determination of the optimal number of latent classes for longitudinal trajectories of lower respiratory syndrome.** **(A)** The Bayesian Information Criterion (BIC) elbow plot showing the model fit across 1 to 4 latent classes. The BIC value decreases continuously as the number of classes increases. **(B)** The smallest group proportion across different models. The red dashed line indicates the predefined 5% clinical significance threshold. Although models with 3 or 4 classes showed lower BIC values, their smallest subgroups fell below this threshold (< 1%), justifying the selection of the two-class model as the optimal and most robust fit.


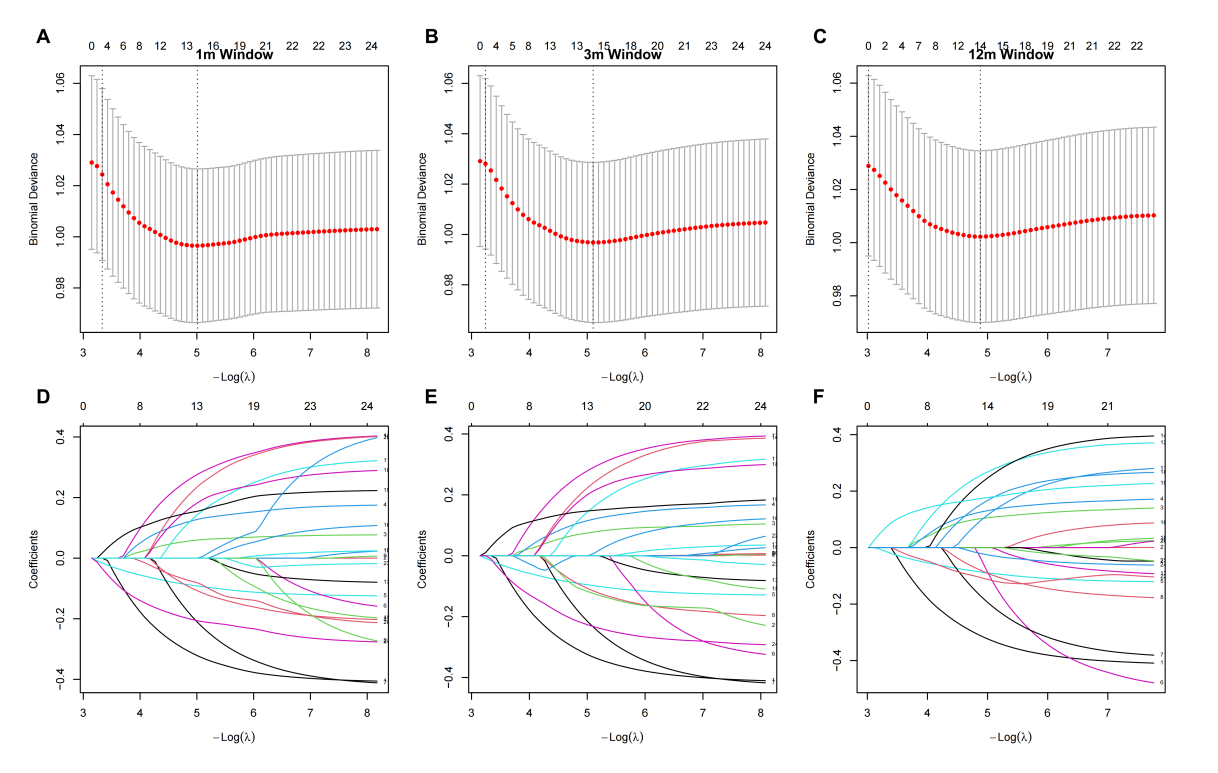


**Figure S2. Feature selection using the Least Absolute Shrinkage and Selection Operator (LASSO) regression across different exposure windows.** **(A–C)** Ten-fold cross-validation error curves for the 1-month, 3-month, and 12-month exposure windows, respectively. The solid vertical lines represent the optimal tuning parameter (λ) that minimizes the binomial deviance, while the dotted vertical lines indicate the λ value at one standard error from the minimum. **(D–F)** The LASSO coefficient profiles of all candidate variables for the 1-month, 3-month, and 12-month windows. A vertical dashed line is drawn at the optimal log(λ) value, corresponding to the selected variables with non-zero coefficients that were subsequently incorporated into the final multivariate models.


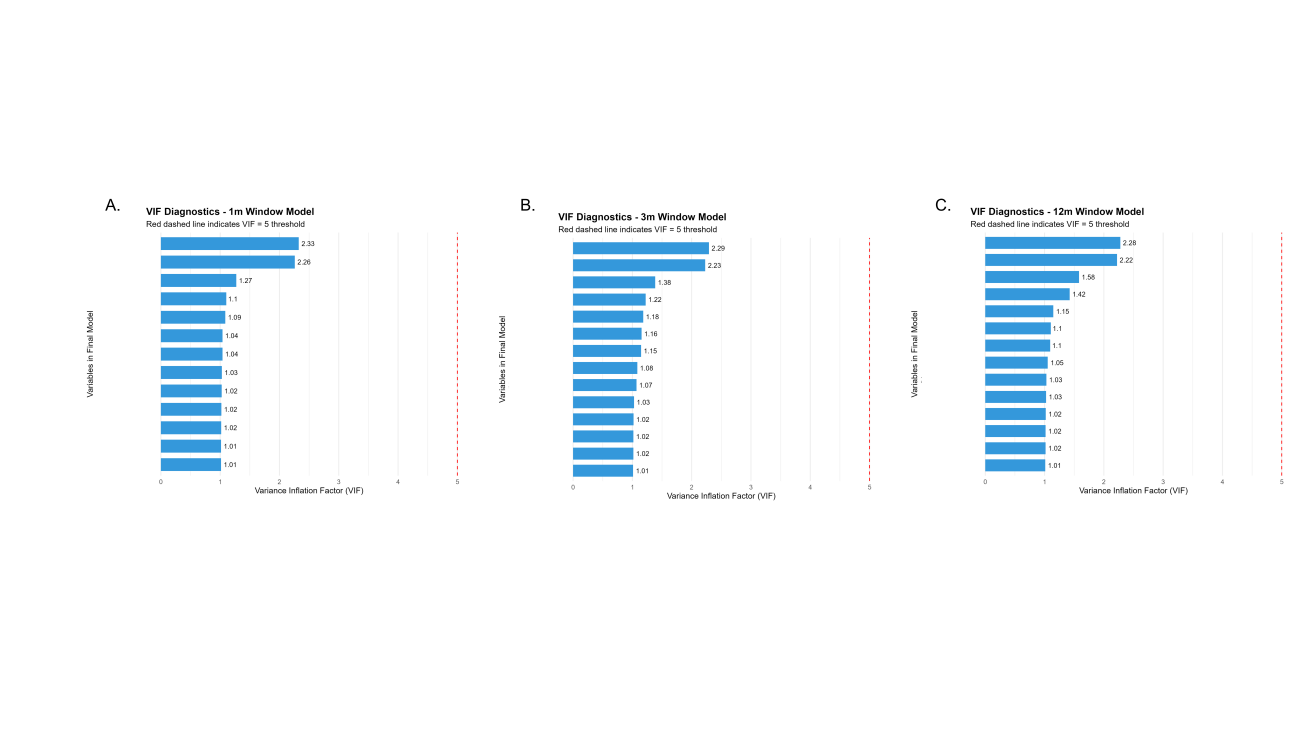


**Figure S3. Assessing multicollinearity among variables using Variance Inflation Factor (VIF) in the final multivariate models across different exposure windows.** **(A–C)** VIF values for all independent variables retained in the 1-month, 3-month, and 12-month exposure window models, respectively. The red dashed line indicates the predefined threshold for severe multicollinearity (VIF = 5). Across all three temporal models, every retained variable exhibits a VIF value well below this threshold (maximum VIF < 2.5), confirming the absence of significant multicollinearity among the predictors.


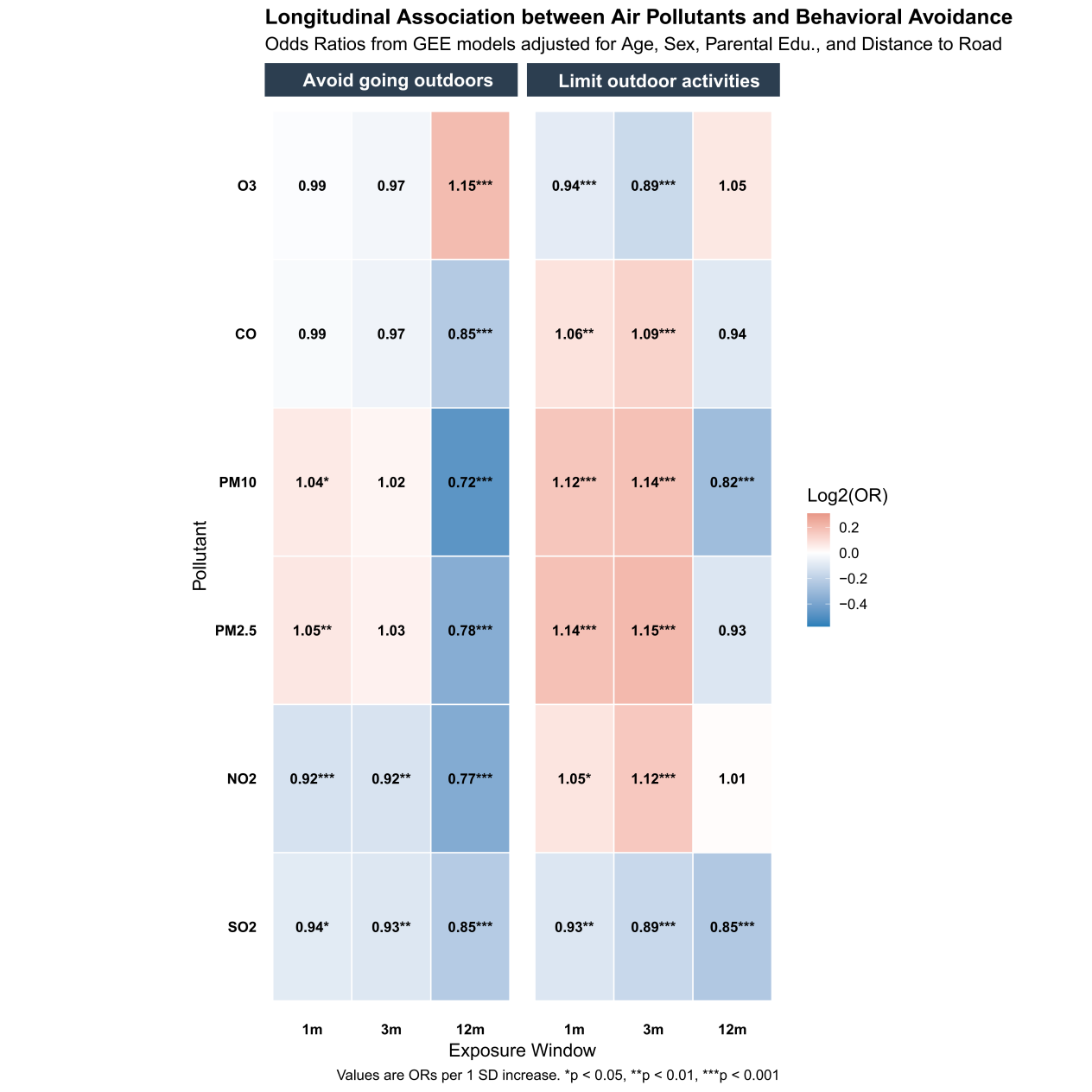


**Figure S4. Longitudinal associations between multi-phase air pollutant exposures and behavioral avoidance.** The heatmap visualizes the Odds Ratios (ORs) derived from Generalized Estimating Equation (GEE) models, adjusting for age, sex, parental education, and distance to the main road. The color gradient reflects the Log2-transformed ORs: warm colors (red) indicate an increased likelihood of adopting protective behaviors (OR > 1), whereas cool colors (blue) signify a decreased likelihood (OR < 1). Actual OR values are displayed within each cell, representing the effect per 1-standard-deviation (SD) increase in pollutant concentration. * *p* < 0.05, ** *p* < 0.01, *** *p* < 0.001.

**
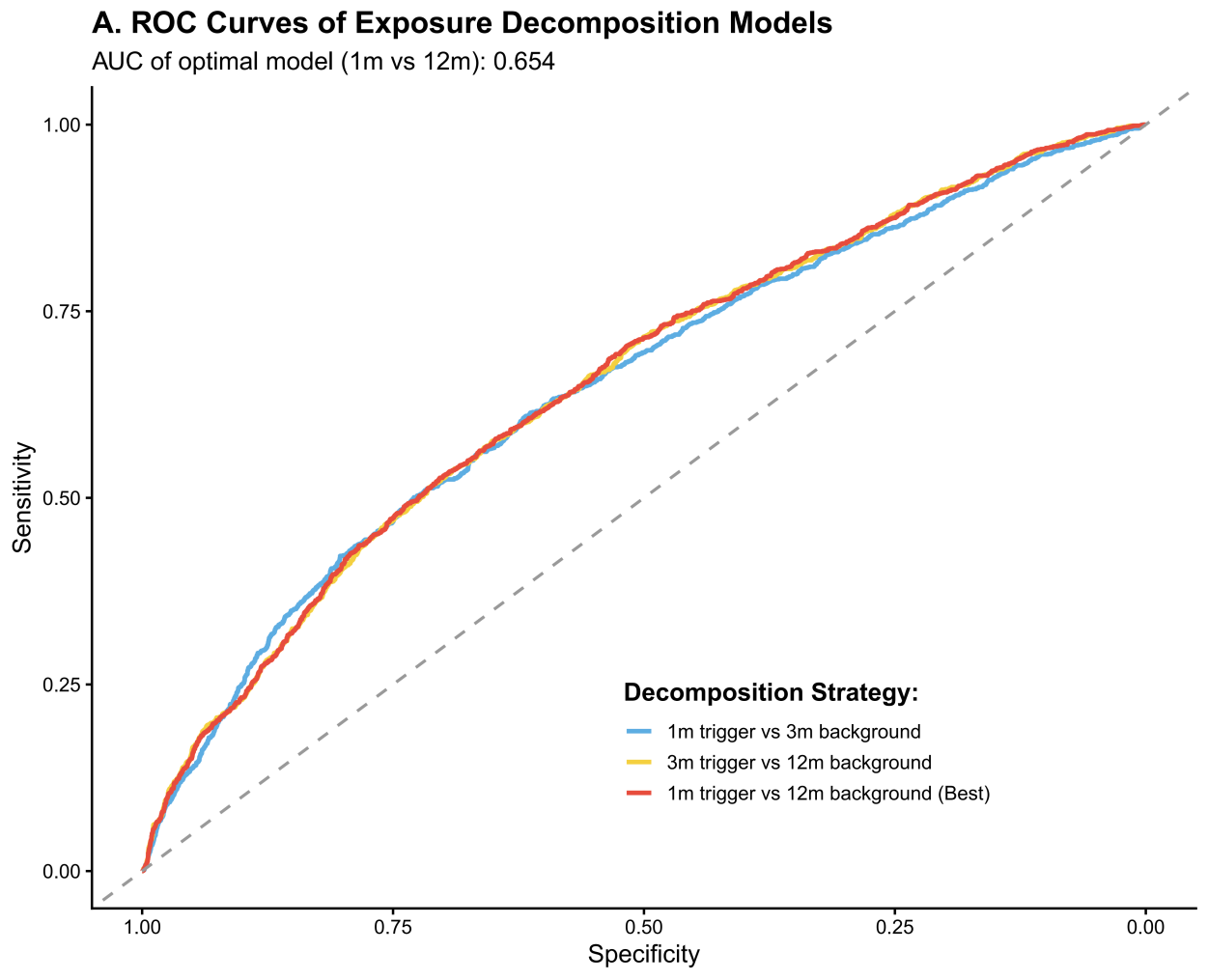
**

**Figure S5. Receiver Operating Characteristic (ROC) curves for different temporal decomposition models.** The plot compares the predictive performance of three exposure decomposition combinations in identifying the persistent moderate risk trajectory: 1-month versus 3-month (1m_vs_3m), 3-month versus 12-month (3m_vs_12m), and 1-month versus 12-month (1m_vs_12m). The combination of the 1-month acute fluctuation and the 12-month chronic background yielded the highest Area Under the Curve (AUC = 0.654), demonstrating its superiority. Consequently, this optimal 1m_vs_12m combination was selected for the subsequent joint effect and threshold analyses.
